# Soil Microbial Communities Changes Along Depth and Contrasting Facing Slopes at the Parque Nacional La Campana, Chile

**DOI:** 10.3390/microorganisms12122487

**Published:** 2024-12-03

**Authors:** Carolina Quinteros-Urquieta, Jean Pierre Francois, Polette Aguilar-Muñoz, Verónica Molina

**Affiliations:** 1 Programa de Doctorado Interdisciplinario en Ciencias Ambientales, Universidad de Playa Ancha, Avenida Leopoldo Carvallo 270, Playa Ancha, Valparaíso 2340000, Chile; carolina.quinteros@alumnos.upla.cl; 2 Departamento de Ciencias y Geografía, Universidad de Playa Ancha, Avenida Leopoldo Carvallo 270, Playa Ancha, Valparaíso 2340000, Chile; geofrancois@gmail.com (J.P.F.); polette.aguilar@upla.cl (P.A.-M.); 3 HUB Ambiental UPLA, Universidad de Playa Ancha, Avenida Leopoldo Carvallo 207, Playa Ancha, Valparaíso 2340000, Chile; 4 Centro de Investigación Oceanográfica COPAS COASTAL, Universidad de Concepción, Concepción 3349001, Chile

**Keywords:** bacteria, archaea, fungi, abundance, ammonia-oxidizing archaea

## Abstract

The Parque Nacional La Campana (PNLC) was recently recognized for its high soil surface microbial richness. Here, we explored the microbial community structure in soil profiles from contrasting facing slopes where sclerophyllous forest (SF) and xerophytic shrubland (XS) develop. Soil physicochemical conditions (dry density, pH, and organic matter C and N isotopic soil signatures) were determined at three depths (5, 10, and 15 cm depths). Amplicon sequencing (16S rRNA and ITS1-5F) and specific quantification (qPCR bacteria, archaea and ammonia-oxidizing archaea, fungi) were used to profile the microbial community. Our results indicate that opposite slopes, with different vegetation types and soil conditions studied potentially explained the spatial variability of the microbial community composition, especially between sites than through soil depth. Discriminative taxa were observed to vary between sites, such as, *C*. nitrososphaera (ammonia-oxidizing archaea) and Sphingomonas, and bacteria associated with Actinobacteria and Bacteroidetes were predominant in SF and XS, respectively. Fungi affiliated with Humicola and Preussia were more abundant in SF, while Cladosporium and Alternaria were in XS. Higher ASV richness was observed in SF compared to XS, for both prokaryotes and fungi. Furthermore, SF showed a higher number of shared ASVs, while XS showed a decrease in unique ASVs in deeper soil layers. In XS, the genus DA101 (Verrucomicrobia) increases with soil depth, reaching higher levels in SF, while Kaistobacter shows the opposite trend. PNLC soils were a reservoir of redundant microbial functions related to biogeochemical cycles, including symbiotic and phytopathogenic fungi. In conclusion, as with the predominant vegetation, the structure and potential function of microbial life in soil profiles were associated with the contrasting the effect of facing slopes as toposequence effects.

## 1. Introduction

Soil microorganisms are essential for biogeochemical cycles due to the decomposition of organic matter and the regulation of greenhouse gasses [1]. Changes in environmental conditions by natural and anthropogenic sources, such as land use and management practices can significantly alter the microbial community structure of the soil, rhizosphere, and plant [2]. Soil microbial communities are highly diverse, consisting of both eukaryotes (algae, fungi, and protozoa) and prokaryotes (bacteria and archaea) [3]. Fungi and bacteria are typically studied separately due to their different metabolic capabilities involved in degrading organic matter and other key ecosystem and ecological functions. For example, fungi have favorable growth in low pH conditions [4,5] and have a greater capacity to degrade recalcitrant organic compounds compared to bacteria [6]. Besides pH, other environmental conditions, such as texture, porosity and humidity, as well as vegetation cover influence soil microbial structure [6,7] and may have a differential effect on soil fungal and bacterial communities [8,9]. On the other hand, disturbances such as fires have the capacity to affect the composition and functions of the soil microbiome, observing in general a decrease in biodiversity [10] and plant–microbiome interactions [11]. 

Physicochemical conditions in the soil are differentially distributed through depth, influencing water retention and availability [12] and, in turn, changing the composition and activity of microbial communities [13]. In fact, soil water content from rainy areas has been reported to promote a high bacterial abundance but low bacterial diversity compared to soils of dry areas [14]. Organic matter distribution and its turnover by microbial community activities are also influenced by sediment depth in temperate zones [15]. Previous studies reported that soils in temperate forests are characterized by the presence of ectomycorrhizal fungal mycelia, which can represent up to one-third of the microbial biomass and produce 50% of the dissolved organic carbon [16]. On the other hand, in dry areas, low microbial abundance has been reported to decrease with depth [17]. Several examples of microbial trait change through soil depth have been reported, such as bacterial diversity that could reach higher values in the top 10 cm of the profile and typically decrease by 20% to 40% towards deeper horizons and composition associated with a decrease in the relative abundance of Bacteroidetes and Verrucomicrobia between 10 and 50 cm [18]. Microbial community composition in the nine pits was more variable in the surface horizons; however, at greater soil depths, communities were relatively similar regardless of landscape position [18].

The soil microbial community structure, including the abundance of bacteria and archaea, was reported to change in response to environmental heterogeneous conditions across different spatial scales from arid to temperate climates of Chile [19,20]. In addition, these reports showed a decrease in the relative microbial abundance of bacteria and archaea with soil depth (0 cm to 40 cm) gradients, which was associated with total organic carbon (TOC) availability [19,20]. Furthermore, a recent report in the north, center, and south of the Chilean Cordillera de la Costa observed changes in the structure of microbial communities involved in pedogenesis along a bioclimatic gradient from north to south [21]. These changes were partly related to climate and environmental factors, such as soil pH, phosphorus availability to plants, organic matter, bulk density, and clay percentage, and were closely linked to the structure and composition of the surrounding vegetation. Moreover, microbial community richness was found to decrease with depth at all southern-facing slope sites and in arid ecosystems on northern-facing slopes.

The relief and Mediterranean climate of central Chile turns this into an ideal area to determine the influence of environmental gradients related to biotic (i.e., vegetation) and abiotic (i.e., facing slope orientation) determinant factors of the landscape potentially shaping the soil microbial diversity [22]. In terms of vegetation, the PNLC shows that its slopes differ according to their slope orientation. The north-facing slopes receive more sunlight and are therefore warmer and drier, favoring the growth of xerophytic shrubland and the Chilean palm. On the other hand, south-facing slopes receive less sunlight, so they maintain cooler and wetter conditions, allowing the development of sclerophyllous forest, and in more humid and elevated areas, vegetation such as deciduous forest [23].

We hypothesize that soil microbial communities and their traits such as abundance and functional potential will vary according to soil depth and sun exposure, reaching low diversity, abundance, and restricted functionality at the north-facing slopes of the park. Therefore, the objective of this study was to determine the soil microbial community structure and specific abundance spatial variability associated with soil depth l (at 5, 10, and 15 cm) at contrasting slope aspect associated with the sclerophyllous forest (south-facing slope) and xerophytic shrubland (north-facing slope).

## 2. Materials and Methods

### 2.1. Study Area and Sampling Collection

**Figure 1:**
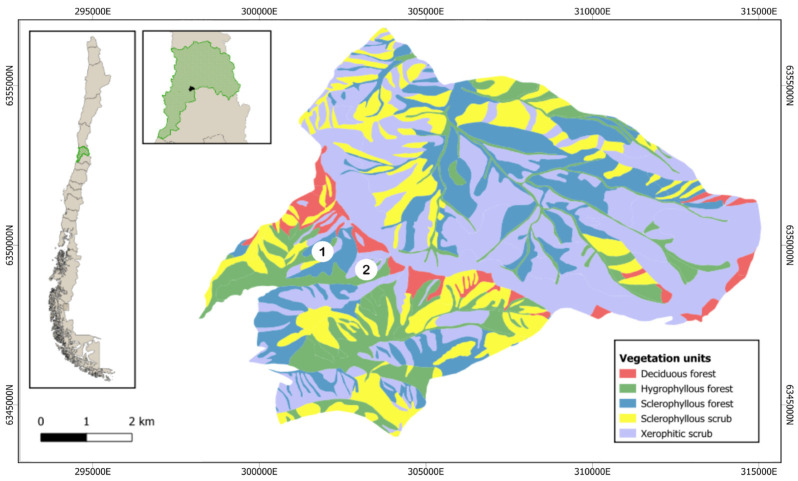
Map that shows PNLC sampling locations (1 = sclerophyllous forest, 2 = xerophytic shrubland) in three depths (5, 10, 15 cm). Vegetation map adapted from Hauck et al. 2016 [23].

**Figure 2:**
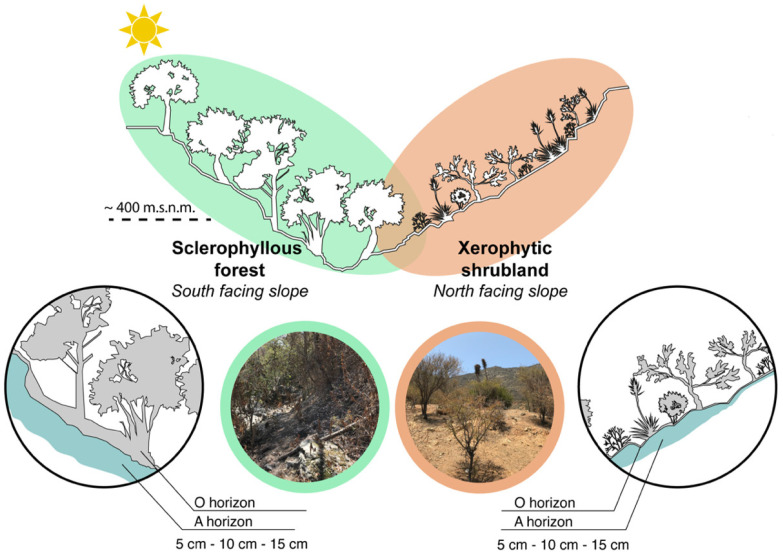
Sampling sites and soil layers.

This study was conducted in the Parque Nacional La Campana (PNLC), an 8000-hectare National Park (1967) and a Biosphere Reserve (1987) area located in the Valparaíso region (32°55′–33°01′ S; 71°09′–71°01′ W, Figure 1 and Figure 2). This area also serves as a crucial reservoir of biodiversity within the Central Chile hotspot [24,25,26]. The landscape is characterized by a relief disrupted by multiple mountains and valleys that are part of the coastal mountain range, and the climate is Mediterranean, with contrasting seasons: hot and dry summers vs. cold and wet winters [27]. This unique combination of topographic and climatic conditions [23] fosters diverse vegetation, including sclerophyllous communities at mid-elevation zones (400–1100 m above sea level). Firstly, sclerophyllous forests including Quillay and Litre forests dominated by *Quillaja saponaria* and *Lithraea caustica*, respectively, and secondly, Xerophytic shrublands constituted by the Matorral de Tebo (*Retanilla trinervia*) were studied. These communities are found on both shady (south-facing) and sunny (north-facing) slopes. Valley floors support hygrophilous communities like the Peumo and Boldo Forest (*Cryptocaria alba* and *Peumus boldus*). Additionally, the park’s northern region harbors palm forests (*Jubaea chilensis*) [28,29]. Higher elevations (1100–2000 m.a.s.l.) are characterized by a vegetation transition to subalpine communities characterized by Roble Forests and Neneo Shrubland (*Nothofagus macrocarpa* and *Mulinum spinosum*) [23,30].

### 2.2. Soil Sampling

The soil sampling was conducted in January 2020 during the austral summer in the Cajón Grande area of the PNLC (Figure 2, Table S1). A total of 20 soil samples were randomly collected from 10 × 10 m plots from 5 cm, 10 cm, and 15 cm depths at an elevation of approximately 400 m.a.s.l. Eight samples were taken from northern-facing slopes, with two sites at only 5 cm depth (because rock was encountered below 5 cm), and 12 from southern-facing slopes, characterized by sclerophyllous forest and xerophytic shrubland, respectively.

Soil samples were collected using sterile 50 mL Falcon tubes, sterile 100 g zip-lock bags, and personal protective equipment, including gloves, to prevent contamination. A clean shovel was also used to collect the samples, and a ruler was employed to ascertain the depth. Then, the samples were transported to the laboratory under dark and cold conditions using gel packs. At the laboratory, the samples were divided into 3 fractions for the different analyses. The sample from the falcon tubes was analyzed for DNA, while the samples from the bags were analyzed for physical and chemical properties. To determine physicochemical soil conditions, 200 g were kept refrigerated at 4 °C for pH and dry density. To determine C and N isotopic analysis, a sub-sample of 50 mg was stored frozen in cryotubes (−20 °C). For molecular analyses, 250 mg was subsampled and preserved in cryotubes with RNA and stored frozen at −80 °C for one month.

### 2.3. Vegetation Coverage and Environmental Conditions in PNLC

The plant species composition and cover were assessed at the sampling sites by evaluating 10 × 10 m plots. The phytosociological methodology [31] was used to conduct a census in each selected area. A list of the species present in each plot was recorded, and their abundance was estimated in terms of the coverage of individuals of each species, expressed as a percentage. The symbol ‘+’ denotes multiple individuals of the same species, while the letter ‘r’ indicates a single individual when abundance was less than 1% [32,33].

The environmental conditions of the sampling sites were obtained from the WorldClim 2.1 database [34]. This included mean annual temperature, maximum temperature of the warmest month, minimum temperature of the coldest month, annual precipitation, precipitation of the wettest month, and precipitation of the driest month. A polygon was defined in the PNLC based on the georeferencing of the sampling points using ArcGis version 9.3 and Global Mapper (see Figure 1 and Table S2).

### 2.4. Soil Physicochemical Analysis

To determine the pH of the soil, 20 g of the dry sample was used in duplicate and resuspended in 50 mL of water. The mixture was stirred with a magnetic stirrer at a controlled temperature of 23 °C for 5 min and allowed to stand for 3 h. The pH was measured using the Thermo Scientific Orion Star A215 X11399 Multiparameter Instrument and Thermo Scientific pH Electrode (Series VZ1-15288) (Thermo Fisher Scientific, Waltham, MA, USA), following the Recommended Methods of Analysis for Chilean Soils [33].

The dry bulk density was estimated using the excavation method [35]. We choose this method, because the alternative ones (e.g., core sampling and clod) cannot be applied to the studied soils by its coarse characteristics with relatively high stone and gravel content [36]. The soil samples were obtained at different depths (5, 10 and 15 cm) and its volume was measured in situ. Later, the samples were weighed and dried at 105 °C for 24 h to remove the water, and the dry-bulk density estimated as the division by the dry weight (quotient) by the in situ measured volume (dividend). 

The composition of soil organic matter was analyzed using an Isotope Ratio Mass Spectrometer (IRMS) Thermo Delta Advantage (Multiparameter Thermo Fisher Scientific, Waltham, MA, USA) coupled to a Flash EA2000 elemental analyzer (Thermo Fisher Scientific, Waltham, MA, USA) at the Applied Biogeochemistry and Stable Isotope Laboratory of the Pontificia Universidad de Chile (LABASI), based on δ15N vs. air and δ13C vs. VPDB values.

### 2.5. DNA Extraction and Sequencing of Phylogenetic Markers of Prokaryotes and Fungi

To analyze the soil microbial community structure, nucleic acids were extracted from 250 mg of soil using the MoBio PowerSoil^®^ kit (MO BIO Laboratories, Inc., Carlsbad, CA, USA). The DNA was quantified using Qubit fluorometer (Thermo Fisher Scientific, Waltham, MA, USA) and spectrophotometrically (Cytation 5) (BioTek instruments, Inc., Winooski, VT, USA). For bacteria and archaea, primers 515F (GTGCCAGCMGCCGCGGTAA) and 806R (GGACTACHVGGGGTWTCTAAT) were used to sequence the V4 region of the 16S rRNA gene [37]. For fungi, the ITS1-5F region was sequenced with primers ITS5-1737F (GGAAGTAAAAGTCGTAACAAGG) and ITS2-2043R (GCTGCGTTCTTCATTCATCGATGC) [38]. Hiseq2500 Illumina sequencing platform (Novogene Biology Information Technology Co., Ltd., Beijing, China) was used for high-throughput sequencing. The resulting sequences were deposited in the ENA PRJEB56868 and PRJEB73862 databases. In the ENA PRJEB56868 database, the sequences associated with the project are A1 = XS1_5, A2 = XS2_5, A5 = XS3_5, A6 = XS4_5, A9 = SF1_5, A12 = SF2_5, A15 = SF3_5, and A18 = SF4_5.

The gene copy number by quantitative Polymerase Chain Reaction (qPCR) of the main domains studied, such as bacteria, archaea and fungi were determined. Moreover, the specific quantification of the functional gene of ammonia monooxygenase (*amoA*) as marker of ammonia-oxidizing archaea (AOA) was also analyzed since it was a discriminant taxon with a well-known function associated with nitrification in topsoil sequencing surveys at PNLC [22]. The qPCR mixture was carried out in a total volume of 20 µL and contained 4.5 µL H_2_O, 10 µL q-PCR master mix, and 0.3 µL ROX (Brilliant II qPCR Master Mix, Agilent Technologies, Santa Clara, CA, USA); the volume of each primer is detailed in Table S3. The thermal program consisted of initial denaturation at 95 °C for 10 min, followed by 40 cycles of denaturation at 95 °C for 30 s, hybridization for 1 min (at a specific temperature for each gene, as indicated in Table S3), and extension at 72 °C for 30 s. Each qPCR run included blanks, calibration standards, and sample in triplicate. Standard curves were prepared between 10^2^–10^7^ copies µL^−1^; efficiency values are reported in Table S3.

### 2.6. Analysis of Microbial Composition and Statistical Analyses

QIIME2 version 2021.4 [37] was used to import and align paired-end Illumina sequencing data from ‘manifest Fastq’ format. The sequences were subjected to quality filtering and chimera checking using the DADA2 denoise-paired plugin in QIIME2, resulting in prokaryotes with lengths ranging from 206 to 312 bp and fungi with lengths ranging from 218 to 424 bp. Details of this curation procedure are shown in Table S4.

Sequences were taxonomically classified into Amplicon Sequence Variant (ASV) using the 13_8 weighted Greengenes database with a 99% similarity level for the 515F/806R sequence region [39]. The UNITE QIIME release for fungi database was used to classify fungi based on ITS regions, also with a 99% threshold [40].

The microbial community was analyzed using R v4.3.1 in RStudio v4.2.2 [41]. First, the libraries were rarefied using the rarefy_even_depth function and the alpha diversity was estimated using estimate_richness and evenness functions in ’phyloseq’ v1.40.0 [42] and ‘microbiome’ v1.18.0 package [43]. Venn diagrams were drawn using the get_vennlist function of the ‘MicrobiotaProcess’ v1.8.2 package [44] at the ASVs level. The changes in the microbial community at the phylum level were analyzed using the amp_heatmap and amp_boxplot functions of the ‘ampvis2’ v2.7.31 package [45]. The prokaryote community structure at phyla level was analyzed using non-metric multidimensional scaling (NMDS) using ordinate function in the ‘phyloseq’ v1.40.0 package and environmental factors were plotted using the envfit function of the ‘vegan’ v2.6-4 package [46]. A differential ASV analysis was performed using the ‘DESeq2’ v1.36.0 package [47] (padj < 0.01) to identify microorganisms that varied significantly across depth levels. Paired comparison was conducted within different soil depths (i.e., 5 vs. 10 cm, 10 vs. 15 cm and 5 vs. 15 cm). The selected ASVs were filtered, and those whose combined total abundance (across all samples) represented more than 3% of the total ASVs were displayed in a heatmap, using the heatmap.2 functions from the ‘gplots‘ v3.1.3.1 package.

Differences in alpha diversity and qPCR between sites were assessed using one-way analysis of variance (ANOVA) followed by Tukey HSD. Normality and homoscedasticity tests were performed to ensure that the ANOVA assumptions were in place. Using the ’stats’ v4.2.2 package’s aov and TukeyHSD functions. PERMANOVA was used to compare microbial community spatial differences between sites and soil depths, using the adonis2 function of the ’vegan’ v2.6-4 package. The potential functions associated with ASVs in the different samples were inferred using the trans_func function in the ‘microeco’ v1.8.0 package [48] package, using the FAPROTAX [49] database prokaryotes and FUNGuild [50] for fungi. The results were visualized with the plot_spe_func_perc function from the same package.

## 3. Results

### 3.1. Environmental and Soil Physicochemical Conditions

As expected, the study sites are characterized by contrasting environmental conditions, leading to the development of different plant communities. In specific, south-facing slopes with sclerophyll forest (SF) growing, present higher plant cover (85%) than north-facing slopes (60%) where xerophytic scrub (XS) occurs. Also, the more continuous canopies in the SF provide enough shadow that allows a lower soil temperature in comparison to areas where XS are present (Table 1). The slope gradient was steep in both study sites, with values of 25% for XS and 37% for SF.

The soils in the study area resemble the Lo Vásquez soil series (Ultic Haploxeralf), characterized to develop on igneous (granitic) materials, with a pH that is slightly acidic (between 6.0 and 6.6) and a low organic carbon content (mean 1.5%) [51]. Specifically, pH average values of 5.8 and 6.1 and organic carbon of 1.3% and 5.3% were recorded for soils present in the XS and SF, respectively. The physicochemical conditions of the studied soils differed slightly between the areas where xerophytic shrubland and the sclerophyllous forest occur (Figure S1). The soils from both sites presented a slightly higher C/N ratio and a lower dry density with depth increment. Surface soil from the SF was more acidic than xerophytic shrubland, and the pH recorded at 10–15 cm was similar (Table S5). The isotopic composition of soil organic matter, based on the d15N and d13C values, also varies towards depth and between the two study sites (Figure S1d). SF soils presented a higher variability at 5–10 cm in the organic matter composition in both C and N fractionation compared with the XS soils but reached a similar d15N and d13C at 15 cm depth.

### 3.2. Microbial Community Alpha and Beta Diversity Spatial Changes (Site and Depth)

A summary of the number of sequences obtained for the microorganisms present in the soil of the PNLC after carrying out the sequencing and curation process is listed in Table S4). In general, it is observed that a higher number of sequences was obtained for fungi, with an average of 141,104 per sample, compared to 92,778 for bacteria and archaea. Rarefaction curve analyses indicate an adequate recovery of the expected richness (see Supplementary Table S2, Supplementary Figures S2 for prokaryotes and S3 for fungi).

**Figure 3:**
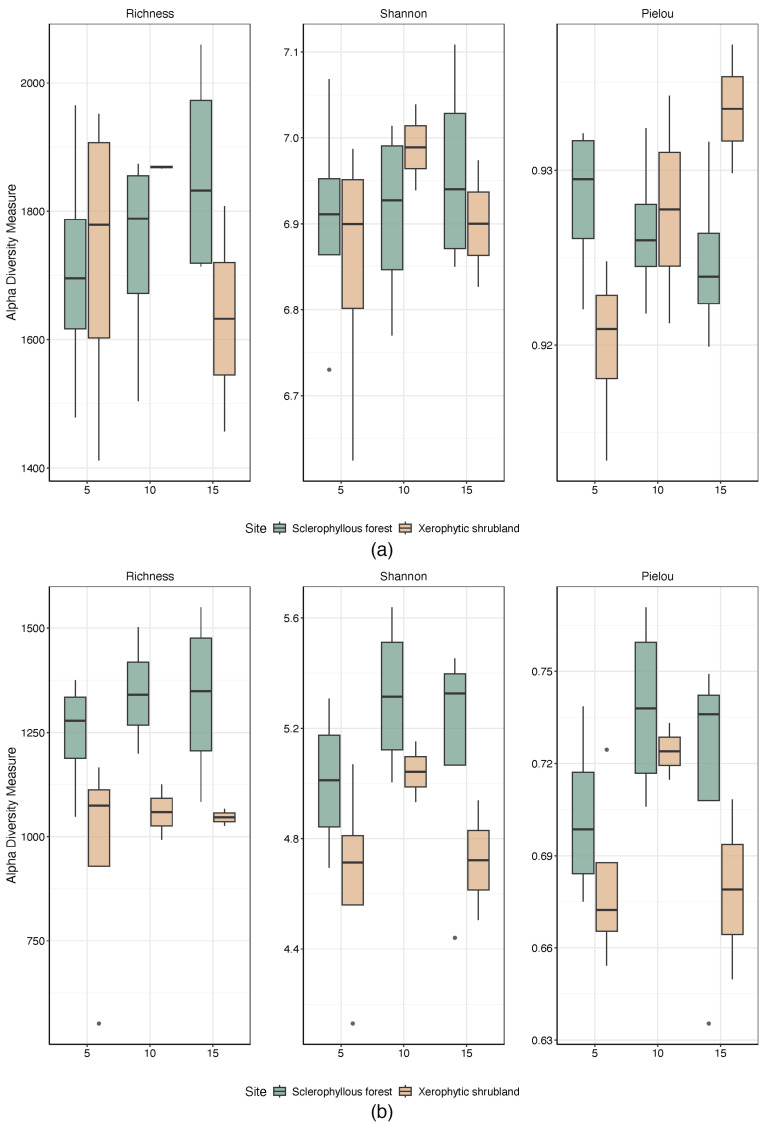
Alpha diversity of soil microorganisms of the sclerophyllous forest and xerophytic shrubland northern and southern slope face of the PNLC at three depths (5–10–15 cm) for (**a**) prokaryotes and (**b**) fungi.

Alpha diversity analyses of prokaryotes indicate that the highest richness is observed in SF (Figure 3a), but only evenness was significantly different at topsoil (5 cm, Pielou index ANOVA *p* < 0.05. Richness and diversity increase with depth in the SF, while evenness decreases with depth. The opposite was observed at the XS soils, showing a significant increment of evenness with depth (5 cm vs. 15 cm, Pielou index, ANOVA *p* = 0.034). For fungi (Figure 3b), SF soils showed higher, but not statistically significant differences in alpha diversity indexes compared with XS (ANOVA, *p* > 0.05) at the three depths evaluated (5, 10 and 15 cm). 

**Figure 4:**
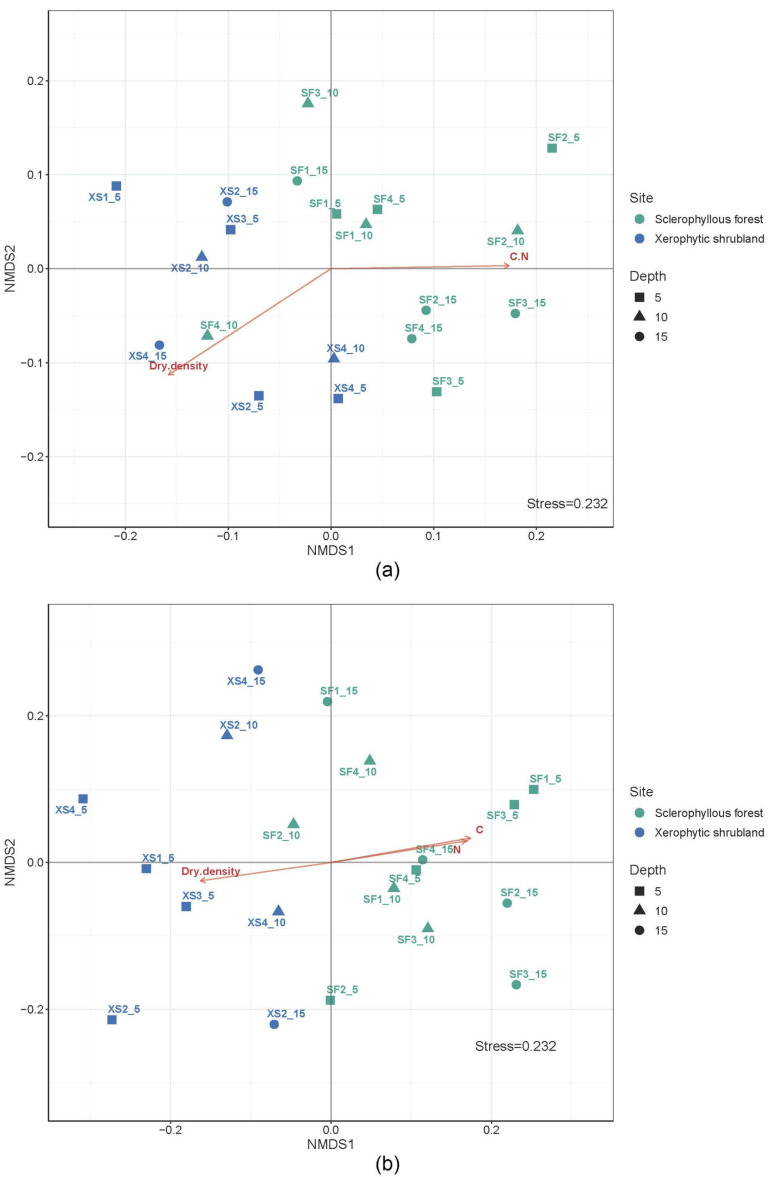
Non-metric multidimensional scaling (NMDS) showing the microbial community structure variability of different soil layers determined at the sclerophyllous forest and xerophytic shrubland of (**a**) prokaryotes and (**b**) fungi.

The microbial community soil profiles comparison between SF and XS were significantly different at ASV level considering prokaryotes and fungi (PERMANOVA *p* = 0.0001). In addition, the comparison of prokaryotes and fungi communities at the ASV level in SF and XS between the different soil layers showed no significant differences (PERMANOVA, *p* > 0.05). In fact, the beta diversity analysis at the phyla level for prokaryotes and fungi are shown by the NMDS (Figure 4a,b). The result of this analysis indicates that the microbial community was more differentiated at the different sites—slopes than at the soil depth layers. Dry density and C:N were associated with the microbial community structure variability. 

**Figure 5:**
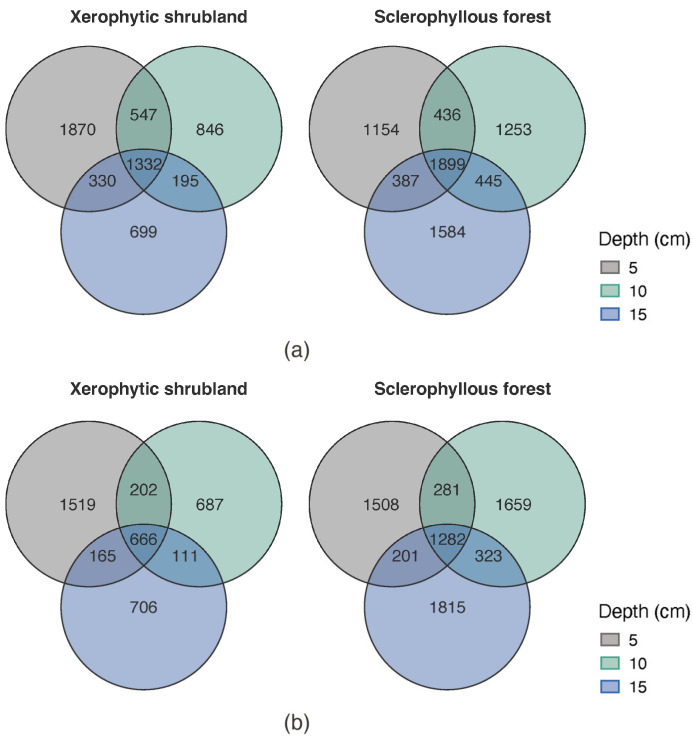
Venn diagram showing the shared and exclusive ASVs associated with (**a**) prokaryotes and (**b**) fungi, grouped according to the soil depth layers determined in the sclerophyllous forest and xerophytic shrubland.

In general, a higher ASV richness was observed at the SF than XS, both for prokaryotes (7158 vs. 5819) and fungi (7069 vs. 4056). The Venn diagram comparing the microbiome richness of prokaryotes (Figure 5a) indicates that the SF soil depths shared a higher number of ASVs compared with the XS, representing 26.52% (1899 ASVs) and 22.89% (1332 ASVs) of the total ASVs (in the three depths), respectively. Compared to prokaryotes, fungi shared a lower number of ASVs in both, the XS (666 ASVs) and the SF (1282 ASVs) accounting for 16.4% and 18.13% of the total, respectively (Figure 5b). In addition, the number of exclusive ASVs of prokaryotes and fungi was characterized by a decrease in the deeper soil depth layer at the XS, whereas the opposite tendency was observed at SF for prokaryotes and fungi.

### 3.3. Microbial Community Composition Spatial Changes (Depth and Sites)

The prokaryote community from the SF soil (Figure S4a) was primarily composed of more than 10% the Proteobacteria, Actinobacteria, and Acidobacteria phyla, which accounted for over 10% of the community, followed by smaller contributions from Verrucomicrobia, Gemmatimonadetes, and other phyla. Actinobacteria, Acidobacteria, and Crenarchaeota were more abundant in the deeper soil layers, while the amount of Proteobacteria and Gemmatimonadetes decreased with soil depth. In contrast, the XS soil community (Figure S4b) was similarly dominated by Actinobacteria, Proteobacteria, and Acidobacteria, but with a higher proportion of Chloroflexi and Verrucomicrobia. Deeper layers of the XS soil showed an increase in Actinobacteria, Verrucomicrobia, and AD3, while Proteobacteria and Acidobacteria decreased. Regarding fungi (Figure S5a,b), Ascomycota was the most abundant phylum in the SF soil, followed by Basidiomycota and Mortierellomycota, which were enriched in deeper samples. Chytridiomycota and Glomeromycota were less abundant. In XS, the fungal community showed a similar dominance of Ascomycota, Basidiomycota, and Mortierellomycota, though Mucoromycota was rare, and Olpidiomycota appeared as a rare taxon. 

**Figure 6:**
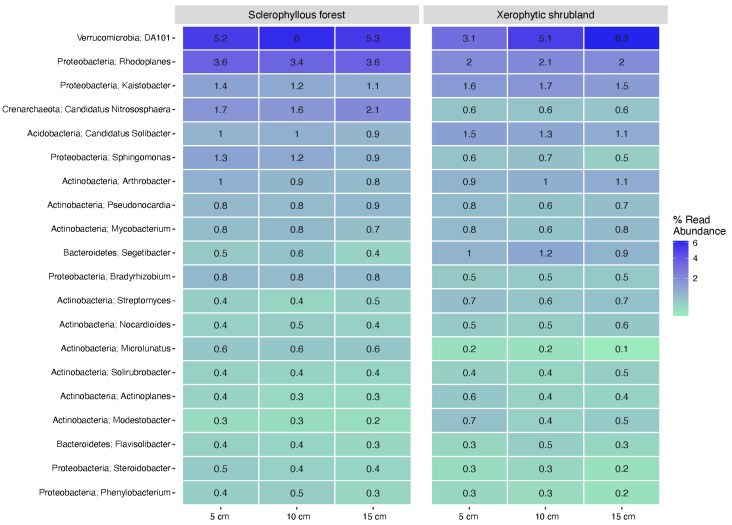
Heatmap of the 20-most abundant prokaryotes genera distribution in three soil depth layers (5 cm, 10 cm, 15 cm) determined in the sclerophyllous forest and xerophytic shrubland.

The heatmap in Figure 6 illustrates the specific genera that varied in abundance within the different soil layers for prokaryotes. For example, in XS, the genus DA101 (Verrucomicrobia phylum) was characterized by its abundance increment with soil depth. An opposite tendency was observed for the genera Kaistobacter for SF (Figure 6). Microbial communities were predominant in one of the sites compared with the other; for example, *C*. nitrososphaera (ammonia-oxidizing archaea) and Sphingomonas were potentially enriched in SF compared with XS, whereas other genera from Actinobacteria and Bacteroidetes phyla were predominantly enriched in XS soil.

**Figure 7:**
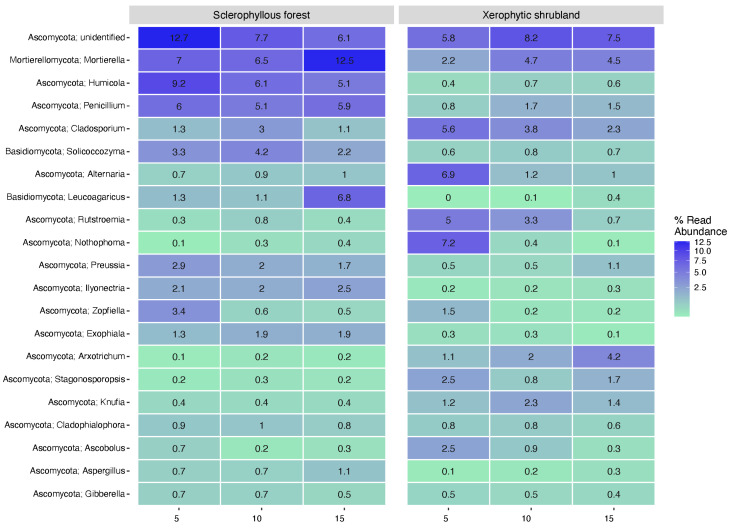
Heatmap of the 20-most abundant fungi genera distribution in three soil depth layers (5 cm, 10 cm, 15 cm) determined in the sclerophyllous forest and xerophytic shrubland.

The heatmap in Figure 7 illustrates the fungal community in the SF and XS soil. In SF, an unidentified genus of the Ascomycota, followed by Mortierella, was predominantly present, showing a decreasing and increasing contribution at deeper portions of the soil, respectively. A differential contribution towards the soil depth studied or a high contribution at the soil surface (5 cm) was observed for many genera, like Humicola, Preussia, and Zopfiella at SF and Cladosporium, Alternaria, Rutstroemia, Nothophoma, Zopfiella, Stagonosporopsis, and Ascobolus at XS (Figure 7). For the opposite trend, the enrichment of specific genera was also determined for a few genera like Leucoagaricus at both the SF and XS soils and Arxotrichum at XS (Figure 7). 

Indicative analyzed taxa were carried out to identify specific discriminant ASV of the soil microbial community comparing northern and southern slopes profiles (Supplementary Figures S6 and S7, prokaryote and fungi, respectively). Prokaryotes exhibit a clustering of more than 50 ASVs distributed in distinct clusters separating XS and SF soil microbial communities. Most of the discriminant ASVs were associated with abundant phyla, mainly related to Actinobacteria (49%). Fungi presented a low number of differentially distributed ASVs per contrasting slopes at XS compared with SF (Supplementary Figure S7). For fungi, phyla sub clustering was mainly associated with soil deeper samples in the SF and surface groups in the XS. 

### 3.4. Microbial Community Abundance and Functional Potential Traits with Depth

**Figure 8:**
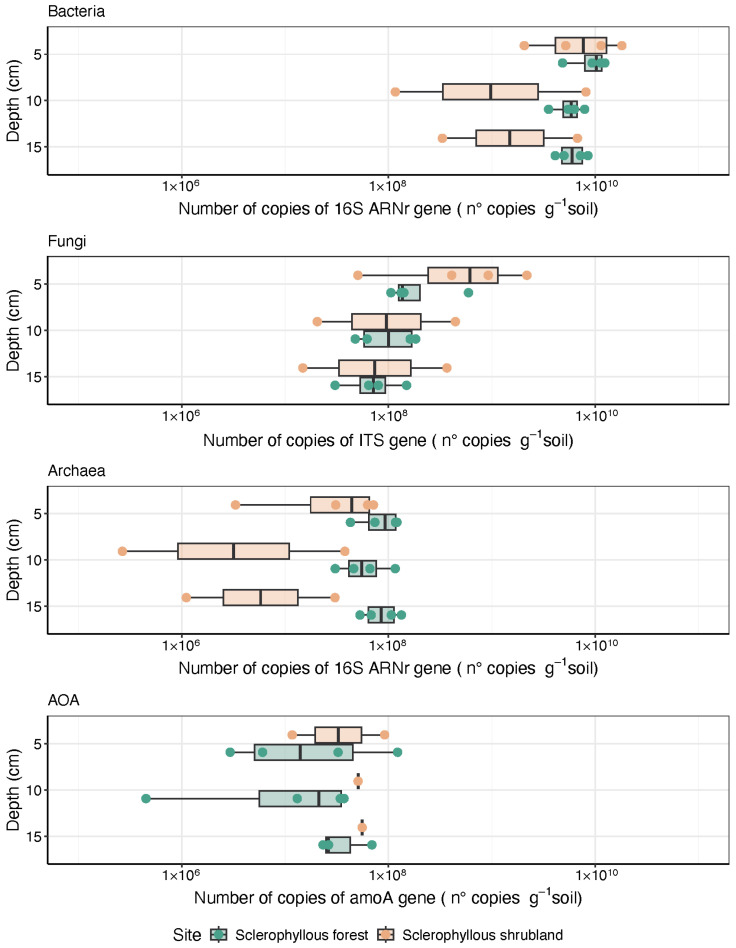
Specific quantification via qPCR of (**a**) bacteria, (**b**) fungi, (**c**) archaea, (**d**) AOA in soil profiles (5 cm, 10 cm, 15 cm depth) determined at the sclerophyllous forest and xerophytic shrubland.

The abundance of specific microbial groups analyzed through qPCR indicates a high variability among the replicates of XS compared with SF, but a similar tendency towards soil depth layers. Bacteria and fungi copies of 16S rRNA genes and ITS, respectively, per DNA ng, presented a tendency to decrease towards depth (Figure 8a,b). Archaea were present at similar levels at all depths, with a potentially higher abundance towards deeper layers in the SF compared with the XS (Figure 8c). These values were comparable with functional gene quantification associated with archaea ammonia oxidizer (AOA) encoding for an ammonia monooxygenase A subunit (Figure 8d). These tendencies were not statistically significant (ANOVA *p* > 0.05).

The prokaryote communities exhibit a similar potential functional profile at each site and depth determined through phylogenetic prediction (Figures S8 and S9). As expected for soil with a high organic matter content, energy was mainly associated with aerobic chemoheterotrophy but also with anaerobic processes that predominate in SF compared with XS, including nitrate reduction and fermentation. Nitrification and specific processes such as aerobic ammonia and nitrite oxidation were also predicted (Figure S8). Regarding the fungi saprotrophic fungi, wood saprotrophs were especially predominant (Figure S9), followed by ecological guilds associated with symbionts such as endophytes and plant and animal pathogens. 

## 4. Discussion

### 4.1. Spatial Changes in the Microbial Community Composition 

The PNLC landscapes were characterized by vegetation known to be adapted to sunlight and water availability, which varies depending on the orientation of the slopes. As previously reported, sclerophyllous forest (SF) and xerophytic shrubland (XS) plant communities were distributed on northern and southern slopes, respectively [23,30]. Besides vegetation, edaphic properties such as physicochemical conditions, temperature, water availability, nutrient dynamics, and microbial activity have been reported to be influenced by the exposure to solar radiation [52,53,54].

Our study supports previous reports on the composition of soil microbial community at the PNLC, characterized by dominant bacterial phyla, such as Proteobacteria, Actinobacteria, and Acidobacteria, as well as the phylum Ascomycota for fungi [21,22]. However, changes in the microbial community structure, especially of dominant prokaryotes, like the prevalence of Actinobacteria over Proteobacteria, were observed in the XS compared to the SF, changes that are in agreement with studies showing the predominance of Actinobacteria in less fertile soils [55]. A comparison of prokaryotic phyla, determined here with other microbial community surveys using similar amplicon sequencing approaches in soil profiles from the PNLC, was included in Table S6. This comparison indicates that although similar core microbial communities were observed, shifts associated with a lower and higher contribution of Planctomycetes and Actinobacteria, respectively, were evidenced in our study compared with previous research [19,21]. In addition, our results agreed with the increasing trends in Acidobacteria, Actinobacteria, and Proteobacteria with sediment depth only at northern slopes, whereas contrasting results were observed for southern slopes.

In addition, also less abundant and rare taxa at phyla level was also preferentially observed at some sites, for example, Crenarchaeaota in the SF, possibly associated with higher humidity [56] and Chloroflexi in the XS, photosynthetic phyla that could be favored by greater sunlight [57]. In addition, Bacteroidetes and Verrucomicrobia were also showed in both sites but showing slightly higher contribution at SF. These taxa have been reported to be common in the rhizosphere and associated with decomposing organic matter [58,59] and were correlated with soil physicochemical properties such as pH and the carbon/nitrogen ratio [60]. Additionally, the phylum Gemmatimonadetes, which was more abundant in the XS, has been reported in xeric soils with a low water content [61].

Fungal community structure was comparable in the SF and XS, with the dominance of Ascomycota and Basidiomycota in the soils of PNLC. Ascomycota has been reported to include a plethora of essential functional contributions to the soil, including organic matter decomposition, humus formation, and nutrient recycling, besides the ecological role associated with mutualistic and parasitic relationships [62,63]. Basidiomycota, on the other hand, can act as saprophytes in grass/forest litter, as wood and yeast decomposers, as ectomycorrhizal fungi, and as plant parasites [64]. Fungi like Glomeromycota were slightly more abundant in the XS compared with the SF, these fungi correspond to a cosmopolitan arbuscular mycorrhizal fungus playing an important role in the ecology and physiology of terrestrial plants [65]. The phylum Chytridiomycota that was predominant in XS soils, has been found in semiarid soils, potentially tolerant to extreme temperatures [66,67]. On the other hand, Mucoromycota, a saprophytic fungus that inhabits soil and manure [68], was enriched in SF compared to XS soils.

### 4.2. Soil Conditions and Vegetation as Drivers of Microbial Community Spatial Changes Traits in the Soils of PNLC

The selected soil sites (SF and XS) presented slightly different conditions associated with the soil isotopic composition (C and N), pH, and dry bulk density. Soils were less acidic compared to a previous study conducted at the PNLC (Table S7). In general, the northern slopes (XS) have a slightly higher pH compared to the southern slopes (SF) and higher nitrogen content based on C/N ratios (Table S7), possibly attributed to the greater vegetation cover at SF than XS, which in turn enhances organic matter inputs. The microbial community structure was potentially associated with the changes in C and N in the soils of SF and XS, based on our ordination analysis (Figure 4). These results support previous reports focused on environmental conditions as drivers of microbial composition, such as higher carbon concentrations on shady slopes, soil temperature, and organic inputs [56,69].

In general, previous research has shown that there is a decrease in microbial biomass, and that the C/N, C/P, and N/P ratios were associated with reduced moisture as the soil depth increases [18,70]. Previous reports have indicated that microbial enzymatic activity related to the phosphorus, carbon, and nitrogen cycles was more intense in the leaf litter than in the soil surface [71]. During our study, slight nitrogen enrichment was observed in relation to depth. Also, different traits of the microbial community were observed to vary with soil depth; for instance, the abundance of bacteria and archaea was characterized by a decrease (qPCR) in soil depth at the northern and southern slopes, a trend that was reported in a previous study (Table S8) that was potentially related to differences in soil characteristics, nutrient availability, and environmental conditions at the time of sampling. In addition, the specificity of the microbial community based on Venn diagram analyses indicated a reduction in exclusive prokaryote ASVs towards deeper soil layers at XS, whereas the opposite trend was found at SF. These observations were supported by alpha diversity indexes at both sites, which showed slight changes towards depth; this was more clearly observed at SF for fungi, which were enriched in the upper soil layer. Moreover, the abundance of bacteria and fungi was higher on the surface. These results align with previous studies demonstrating changes in bacterial and fungal communities with soil depth [70,72]. High fungal abundance and diversity have been reported in organic matter-rich soils, with 60% of taxa concentrated in the top ten centimeters of the soil profile [73]. Most fungal activity is typically found within the upper 20 cm, where nutrient availability and root interactions are higher [74]. Interestingly, our results also showed an increase in fungi ASVs with depth in both SF and XS soils, suggesting that specific fungal groups may thrive in deeper soil layers, potentially benefiting from unique physicochemical conditions or organic matter inputs at these depths. This depth-dependent fungal distribution highlights the importance of both surface and subsurface layers in supporting diverse microbial communities, likely shaped by a combination of plant inputs and soil structure. However, other factors could influence the distribution of microbial community traits, such as moisture [75] and its combination with top-down controls such as predation, whose activity and mobility are reduced by soil dryness [76]. Moreover, the vegetation canopy, the amount and quality of leaf litter deposited at the topsoil [77], and roots could be significant factors influencing soil microbial processes [78]. Additional studies should be carried out to identify the relevance of the above-mentioned drivers of microbial traits. 

### 4.3. Functional and Ecological Microbial Community Potential in PNLC

The observed patterns in the prediction of microbial metabolic potential and diversity in PNLC soils appear to be influenced by both soil depth and vegetation cover. This is supported by ecological traits like plant–microbe interaction, including symbionts and phytopathogens. For example, Actinoallomurus, Conexibacteraceae and Gaiellaceae (Actinobacteria) in XS have been described mainly in tropical areas [75], which are composed by mesophilic bacteria [79] potentially strict aerobes and that have been proposed to be associated with plants [80]. In fact, in XS, the abundant genera Nothophoma at the topsoil encompasses a variety of plant pathogens [81]. Potentially metabolically diverse fungi were detected at SF at all depths but with a higher contribution at different depth layers, for example, Neonectria and Ilyonectria, including several pathogens that are important to both plants and humans, as well as several species that are widely used as biodegraders and biocontrol agents in industrial and commercial applications [82]. The Didymellaceae family was also found to be relevant at a 10 cm depth characterized by the cosmopolitan order Pleosporales, hosting a wide range of species with diverse functions, including plant pathogens, saprophytes, and endophytes [83]. In fact, Didymellaceae is one of the most diverse families in the fungal kingdom, with a wide range of species found in diverse ecosystems [82,83], consisting of up to 31 genera and more than 5400 recorded species [84]. They cause various diseases on fruits, leaves, stems, and roots of different hosts. In addition, some species act as endophytes or saprophytes, often forming associations with both host and non-host organisms [85,86,87]. The families Nectriaceae and Didymellaceae are closely related as plant pathogens [88]. Didymellaceae is a fungal family that includes phytopathogenic members that primarily affect leaves and stems; common genera in this family include Didymella [89]. These fungi are known to be causal agents of leaf spot diseases and can affect soil structure during litter decomposition [90]. 

In general, the soil of the PNLC included a rich and diverse microbial community found associated with different metabolic potential and redundancy at different depths. The prokaryotic communities predominantly utilize aerobic chemoheterotrophy for energy production, nitrate reduction related to the nitrogen cycle, and cellulolytic and fermentation related to the carbon cycle according to our phylogenetic metabolic prediction analyses (Figure S4). The detection of ammonia-oxidizing archaea in our sequences associated with *C*. nitrososphaera and other Crenarchaea were corroborated by the functional prediction in deeper portions of the soil at XS and SF (Figure 8). Aerobic ammonia oxidation was mainly associated with archaea and was supported by the specific detection of AOA via an *amoA* gene-qPCR approach, which was highly variable at the surface but more consistent in deeper soils at both sites. These results were supported by previous studies showing an increased diversity and abundance of archaea at deep layers [91]. The community structure of AOA and their bacterial counterparts have been closely related to factors such as plant species, temperature, water content, C:N ratio, and total soil nitrogen [92,93]. Studies conducted in arid and semi-arid regions of Israel have shown that the community structures of ammonia oxidizers, including archaea and bacteria, were tolerant to the extreme climatic conditions of desert ecosystems, especially AOA as predominant in drier periods and higher temperatures [94]. 

## 5. Conclusions

The heterogeneity of topographic conditions in the PNLC, associated with elevation and slope exposure, influenced the development of vegetation (i.e., XS and SF plant communities) and the physicochemical conditions in soils. Vegetation was found to be associated with soil physicochemical conditions and microbial community structure, predominantly for prokaryotes, which varied in association with dry density and C and N availability in the PNLC soil. Bacteria associated with Actinobacteria were found to be the predominant discriminating taxa when comparing XS and SF, while structural changes in the fungal community were more distinctive at deeper soil depths (10 and 15 cm). Soil profiles at the scale studied (5–15 cm) evidenced changes in microbial community traits; a higher number of ASVs exclusive to deeper soil layers for SF fungi were detected, and a lower number for XS. The surface soil layer (5 cm) of the PNLC was enriched with an abundant community of bacteria and fungi, but was less diverse compared to the deeper layers, which were characterized by more diverse communities in SF, including the presence of functional groups such as ammonia-oxidizing archaea.

However, not all the variability observed in the vegetation landscape should have been attributed solely to the type of vegetation cover; other subsoil characteristics, such as soil depth, also played an important role and could have been the underlying reason for the variation in vegetation types. This suggested that both physical and biological soil properties should have been considered to fully understand the factors influencing microbial community structure in these ecosystems.

Furthermore, other studies conducted at the PNLC showed variations at the phylum level, as well as pH and C/N values at both depth and site, which could have been associated with nutrient availability or vegetation cover. These observations highlighted the importance of considering both depth and site characteristics when assessing microbial communities in different environmental contexts. The soil of the PNLC showed high metabolic redundancy associated with organic matter and nutrient recycling, including aerobic and anaerobic metabolisms of bacteria and archaea. In addition, ecological traits linked to plant–microbe interactions, such as symbiotic and phytopathogenic interactions, were also detected. Future research should prioritize the investigation of the specific functions of discriminating taxa and the activity of various microorganisms in the soil of the PNLC, including their specific role in the biogeochemical cycles of key ecosystems and the ecological interaction of plants, as well as their possible adaptation to climate change.

## Figures and Tables

**Table 1:** Census of plant species found in the SF and XS and canopy, shrub and grass cover (%).

Site	Species	Coverage	Site	Species	Coverage
SF	*Peumus boldus*	70%	XS	*Vachellia caven*	60%
SF	*Cryptocarya alba*	45%	XS	*Conyza bonariensis*	30%
SF	*Citronella mucronata*	35%	XS	*Retanilla trinervia*	30%
				*Lithraea caustica*	15%
SF	*Quillaja saponaria*	30%	XS	*Puya chilensis*	15%
SF	*Adiantum chilense*	15%	XS	*Trichocereus chiloensis*	5%
SF	*Adenopeltis serrata*	10%	XS	*Senna stipulacea*	2%
SF	*Chusquea cumingii*	+	XS	*Asteraceae indet*	+

The symbol ‘+’ denotes multiple individuals of the same species, while the letter ‘r’ indicates a single individual when abundance was less than 1% [32,33].

## Data Availability

The original contributions presented in this study are included in the article/Supplementary Materials; further inquiries can be directed to the corresponding author.

## Supplementary Materials

The following supporting information can be downloaded at https://www.mdpi.com/article/10.3390/microorganisms12122487/s1: Figure S1: Distribution of physicochemical conditions in the soil profiles: (a) C/N ratios, (b) pH (c) dry density and (d) isotopic signature variability (δ15N y δ13C) determined for SF and XS, Figure S2: Rarefaction curves for prokaryotes, Figure S3: Rarefaction curves for fungi, Figure S4: (a) Box plot of prokaryotes phyla relative abundances determined at sclerophyllous forest, (b) Box plot of prokaryotes phyla relative abundances determined at xerophytic shrubland (b) of the PNLC, Figure S5: (a) Box plot of prokaryotes phyla relative abundances determined at sclerophyllous forest, (b) Box plot of prokaryotes phyla relative abundances determined at xerophytic shrubland (b) of the PNLC, Figure S6: Heatmap of potentially discriminative prokaryote ASVs having a significant differentiation comparing north and south slope faces at three depth levels (5 cm, 10 cm, 15 cm), Figure S7: Heatmap of potentially discriminative fungi ASVs having a significant differentiation comparing north and south slope faces at three depth levels (5 cm, 10 cm, 15 cm), Figure S8: Predicted potential microbial function profile by comparison of XS and SF at three depth levels (5 cm, 10 cm, 15 cm) for prokaryotes, and Figure S9: Predicted potential microbial function profile by com-parison of XS and SF at three depth levels (5 cm, 10 cm, 15 cm) for fungi, Table S1: Specific lo-cation, site characteristics, predominant plant community and other topographic features such as elevation and orientation, Table S2: Environmental conditions of the sampling sites, Table S3: Primers used for the quantification of bacteria, archaea and fungi, and specific amplification conditions for each group, Table S4: Per-sample sequence counts and Table S5: Physicochemical conditions of XS and SF soils, Table S6: Comparison of dominant phyla be-tween studies of northern and southern slopes of the PNLC, Table S7: Comparison of physicochemical conditions between studies of northern and southern slopes of the PNLC and Table S8: Comparison of Bacterial and Archaeal Gene Copy Numbers (g^−1^ soil) between studies of northern and southern slopes of the PNLC.
