# Supplementary figures and images for: Soil Microbial Communities Changes Along Depth and Contrasting Facing Slopes at the Parque Nacional La Campana, Chile
Source: Microorganisms. 2024 Dec 3;12(12):2487. doi: 10.3390/microorganisms12122487 (PMC11728372; doi:10.3390/microorganisms12122487)

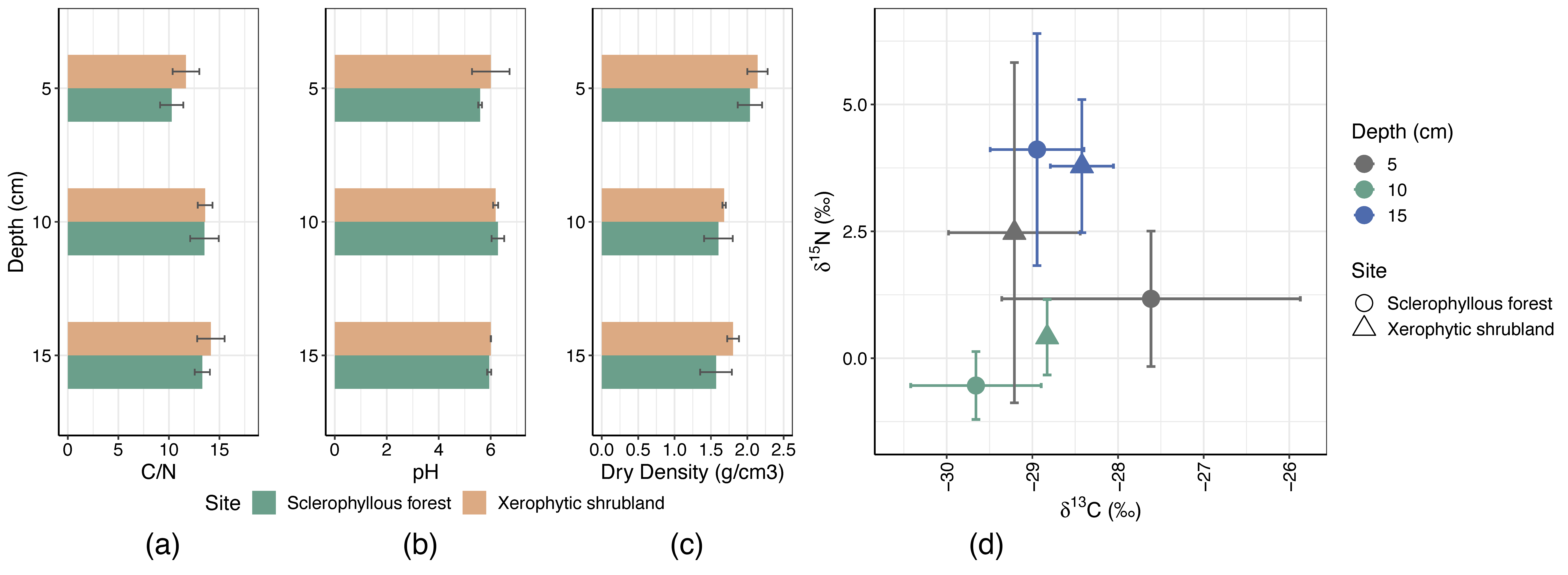

Supplement: Supplementary file 1 [file microorganisms-12-02487-s001.zip › fig S1.tiff]

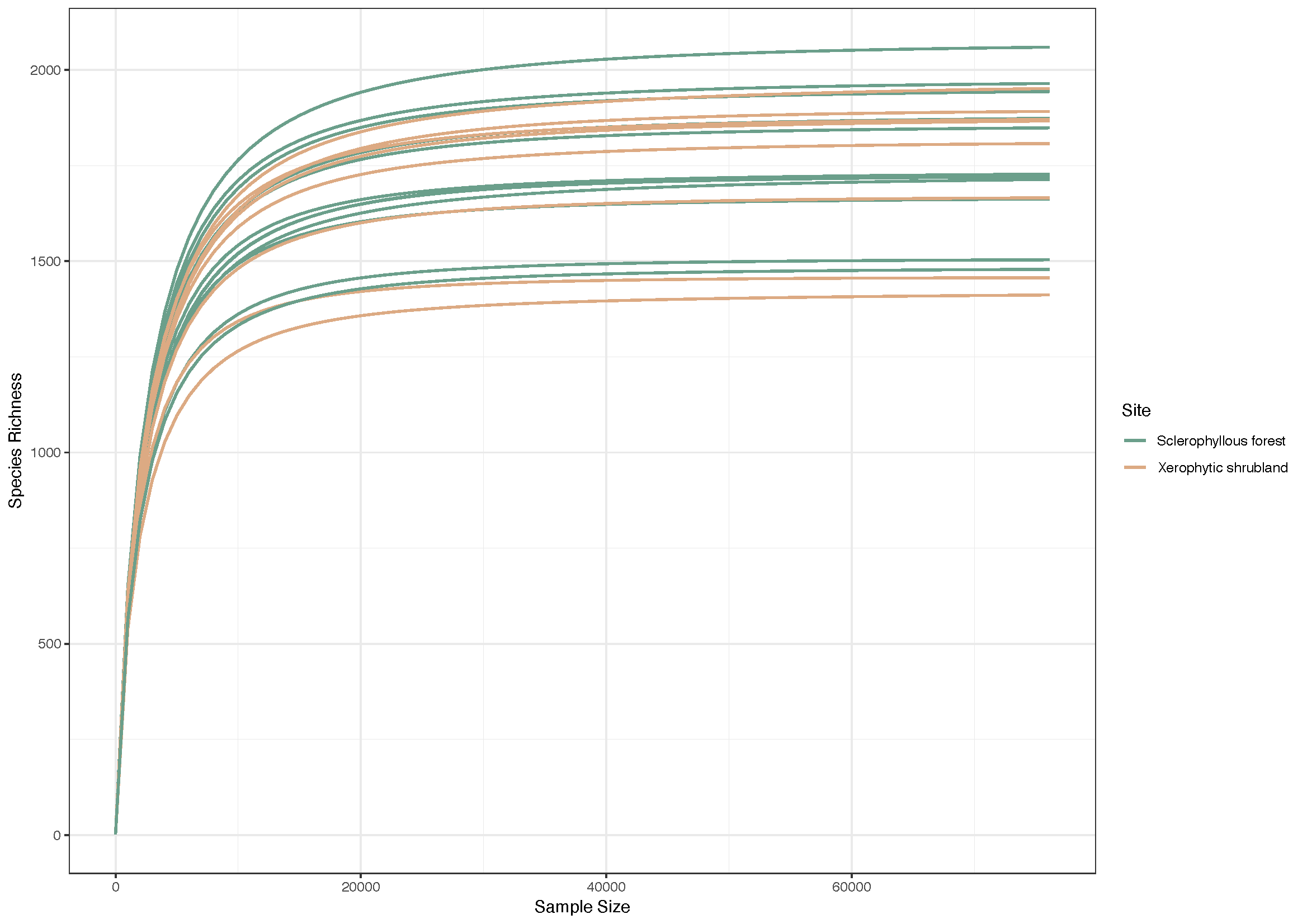

Supplement: Supplementary file 1 [file microorganisms-12-02487-s001.zip › fig S2.tiff]

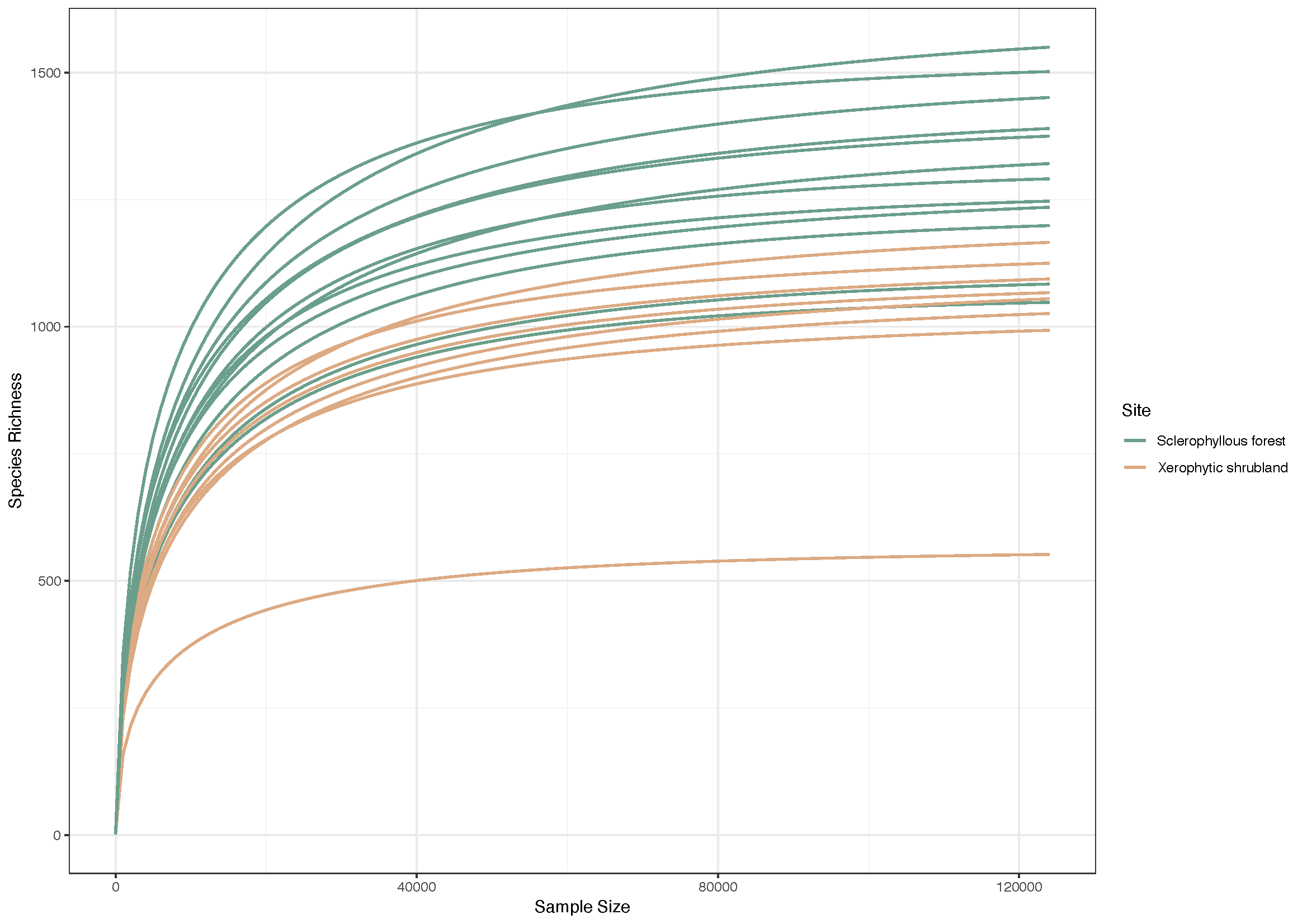

Supplement: Supplementary file 1 [file microorganisms-12-02487-s001.zip › fig S3.tiff]

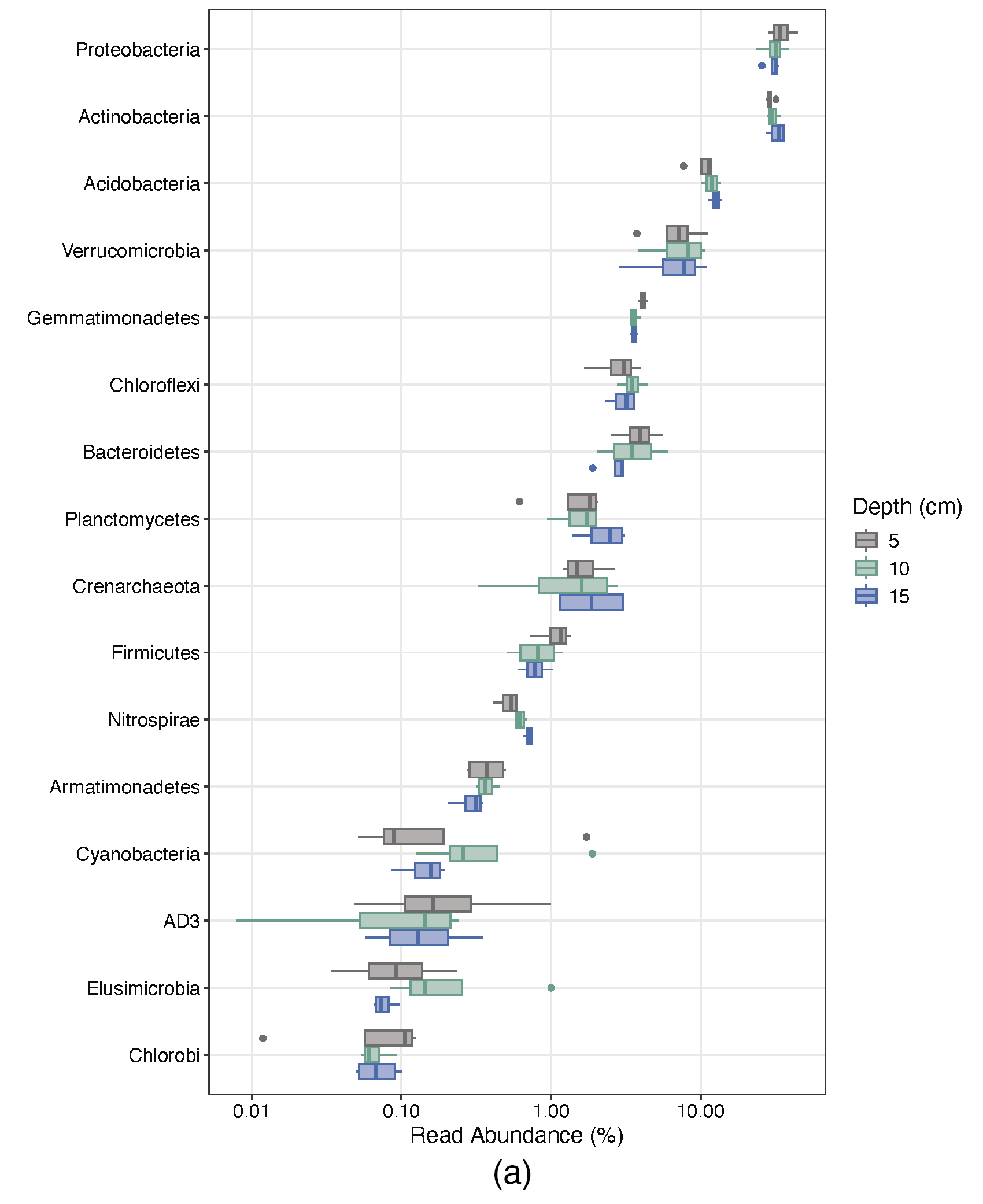

Supplement: Supplementary file 1 [file microorganisms-12-02487-s001.zip › fig S4a.tiff]

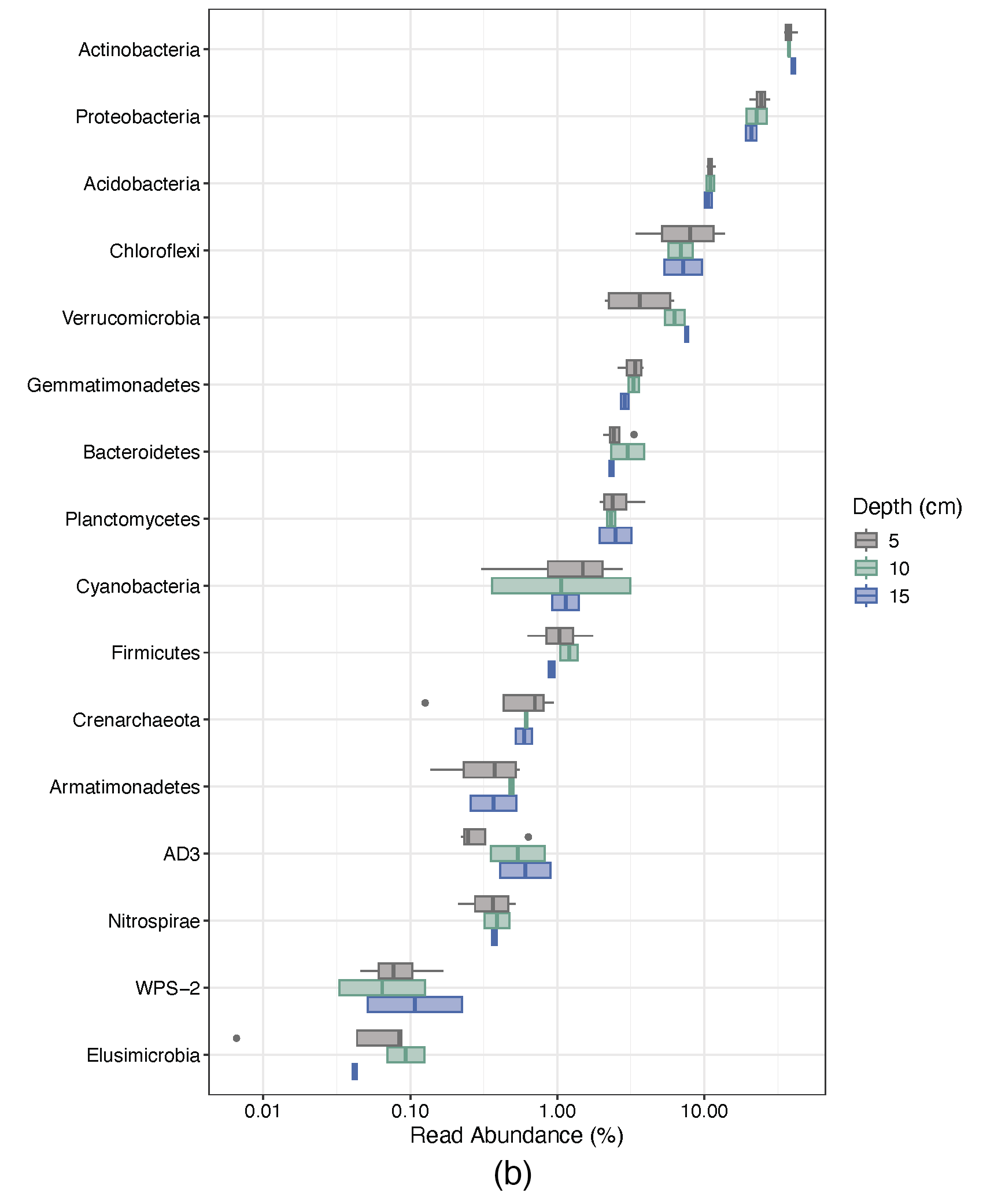

Supplement: Supplementary file 1 [file microorganisms-12-02487-s001.zip › fig S4b.tiff]

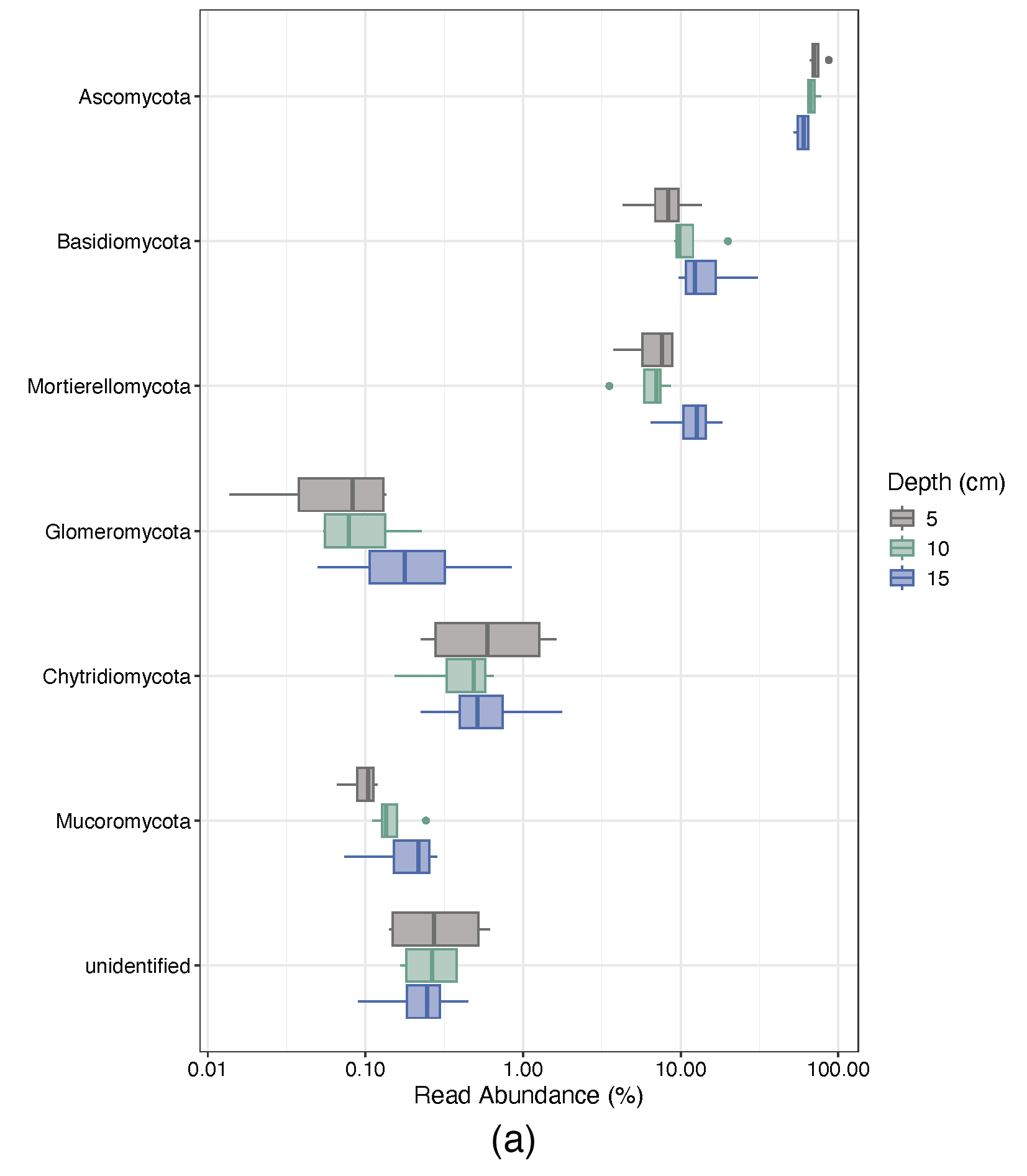

Supplement: Supplementary file 1 [file microorganisms-12-02487-s001.zip › fig S5a.tiff]

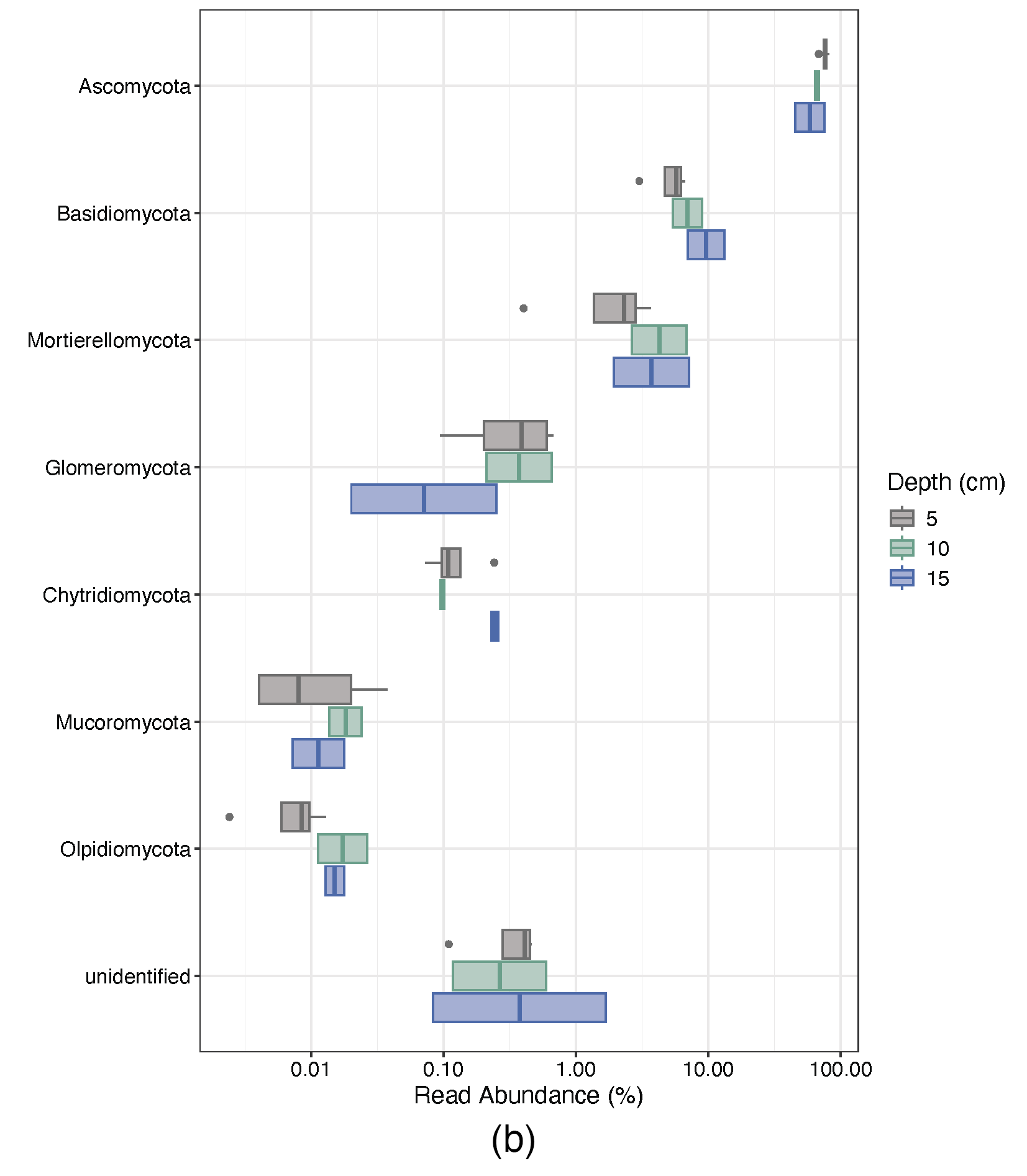

Supplement: Supplementary file 1 [file microorganisms-12-02487-s001.zip › fig S5b.tiff]

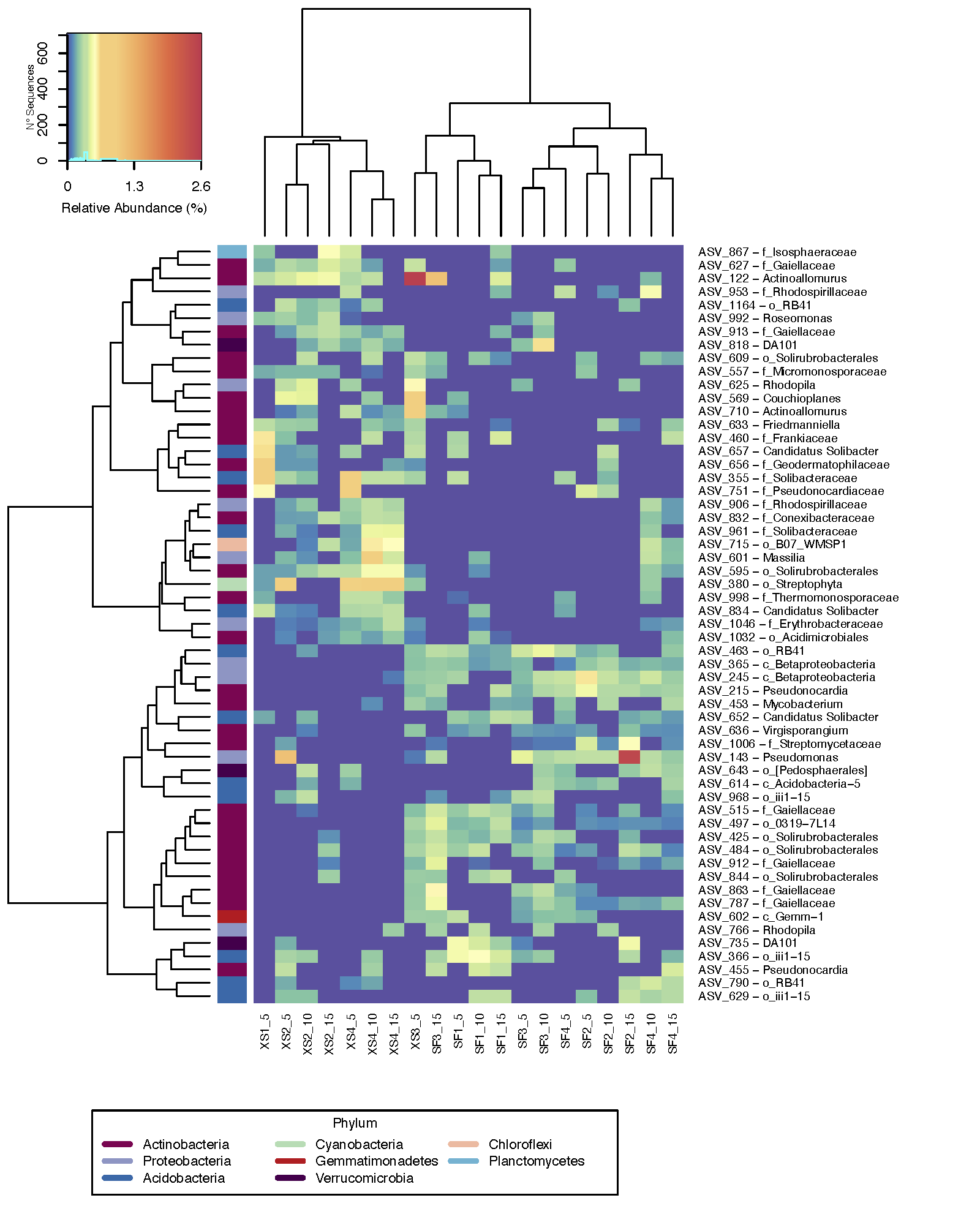

Supplement: Supplementary file 1 [file microorganisms-12-02487-s001.zip › fig S6.tiff]

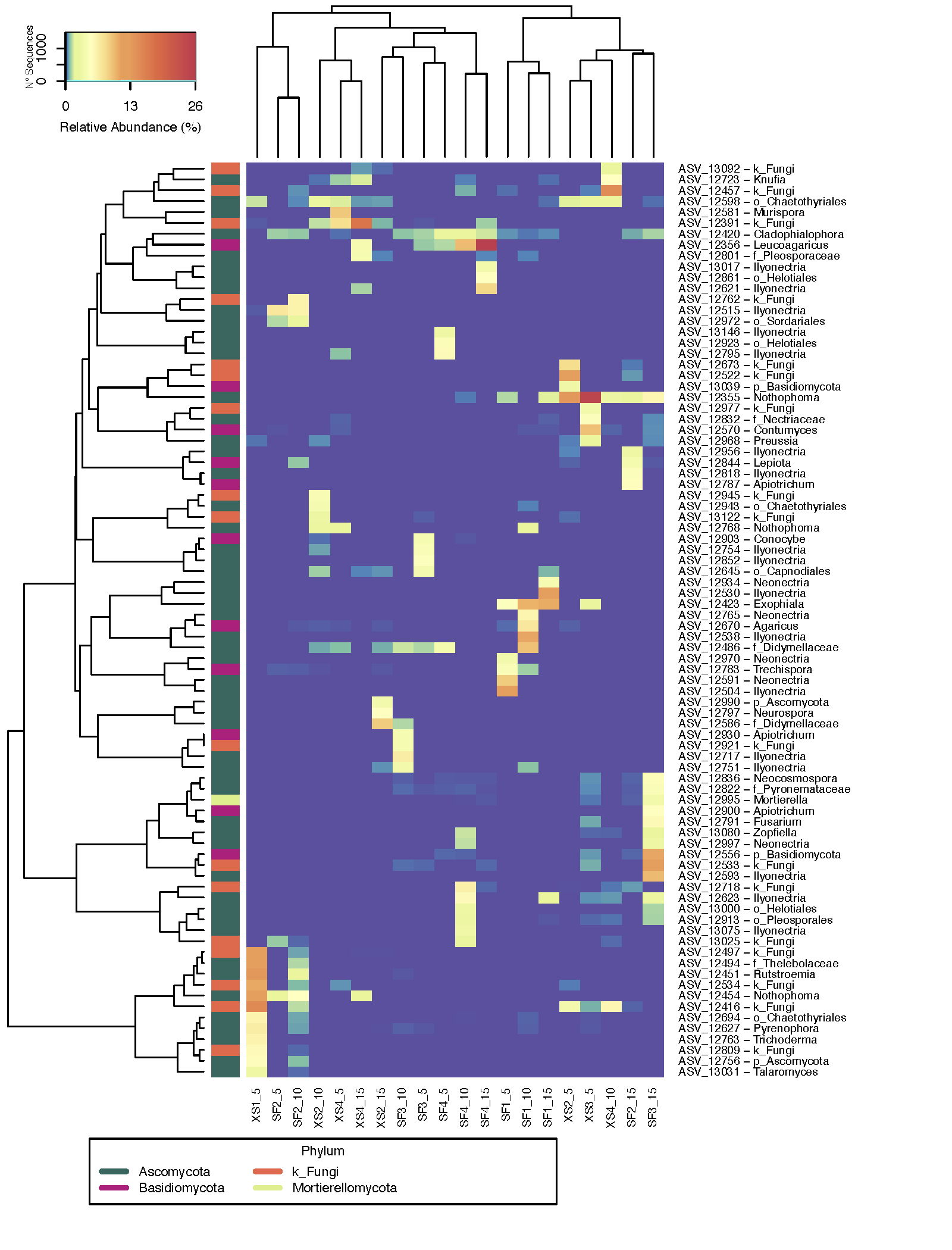

Supplement: Supplementary file 1 [file microorganisms-12-02487-s001.zip › fig S7.tiff]

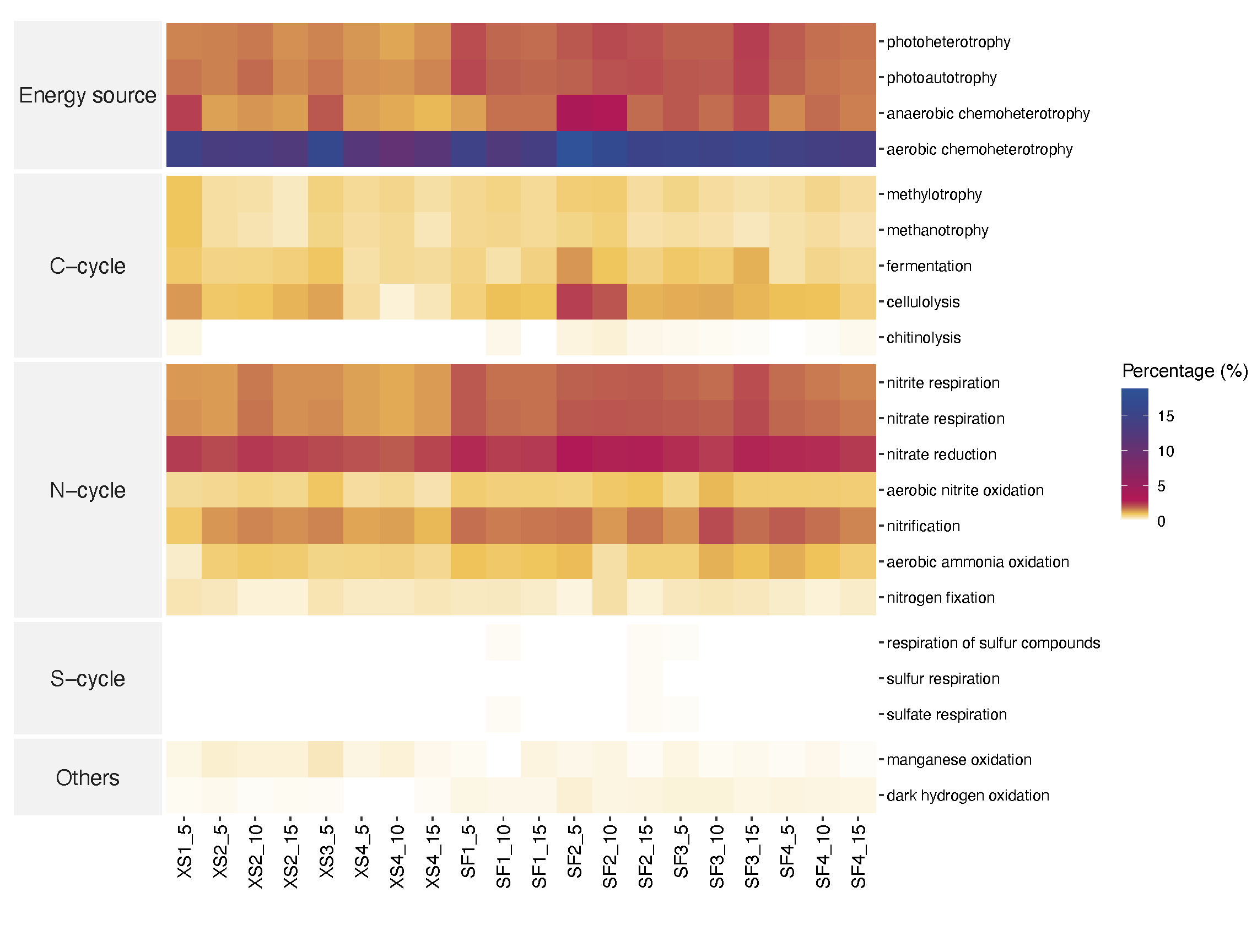

Supplement: Supplementary file 1 [file microorganisms-12-02487-s001.zip › fig S8.tiff]

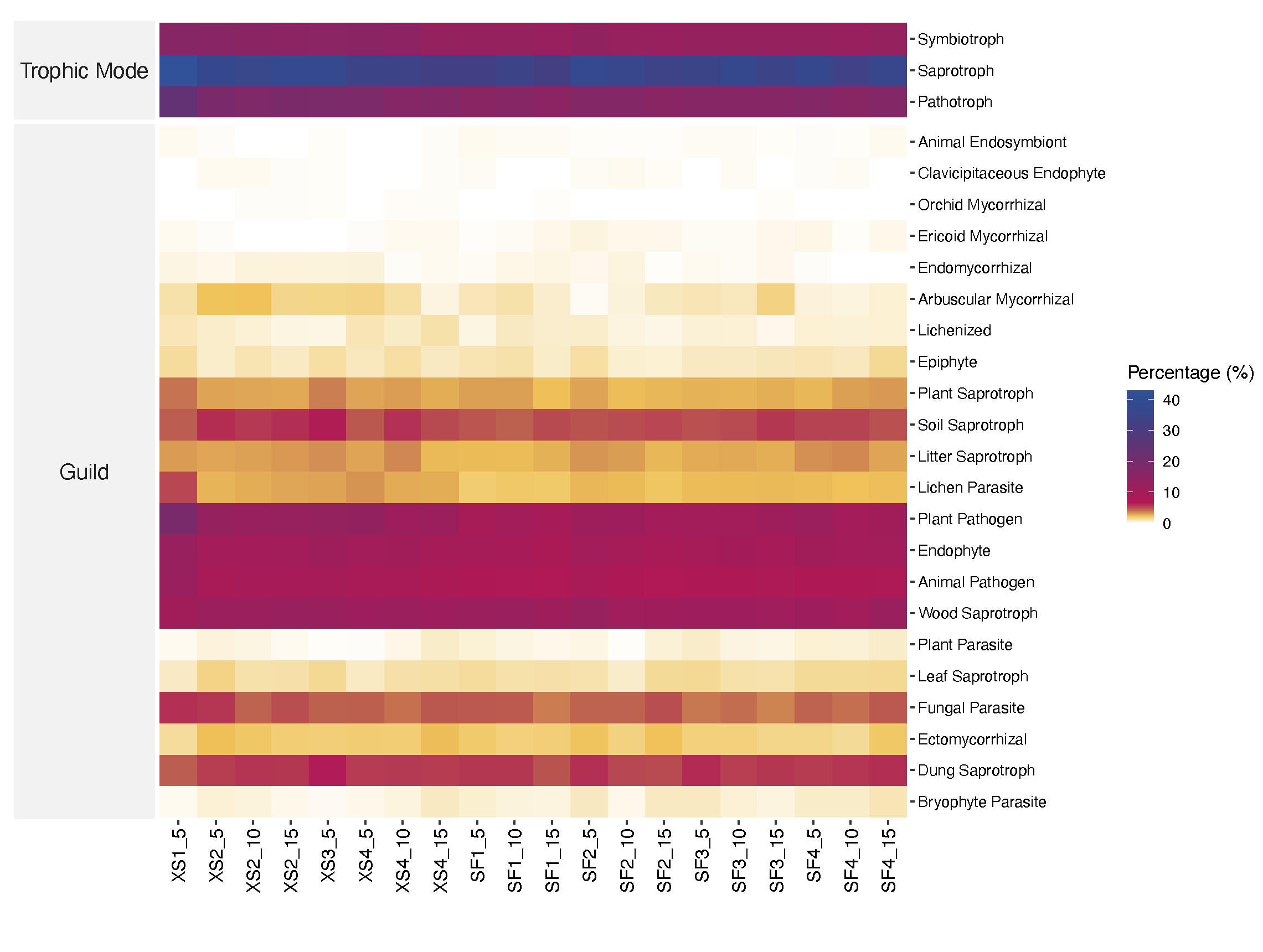

Supplement: Supplementary file 1 [file microorganisms-12-02487-s001.zip › fig S9.tiff]
